# A 17-level quadruple boost switched-capacitor inverter with reduced devices and limited charge current

**DOI:** 10.1038/s41598-024-56717-8

**Published:** 2024-03-14

**Authors:** Majid Hosseinpour, Masoumeh Derakhshandeh, Ali Seifi, Mahdi Shahparasti

**Affiliations:** 1 Department of Electrical and Computer Engineering, University of Mohaghegh Ardabili, Ardabil, Iran; 2 School of Technology and Innovations, University of Vaasa, Vaasa, Finland

**Keywords:** Electrical and electronic engineering, Solar energy

## Abstract

In this paper, a quadruple boost switched-capacitor multi-level inverter is proposed. The proposed structure utilizes a DC source, 11 switches, and a diode to achieve 17-level output voltage levels. This structure consists of three capacitors with the ability for self-balancing voltages. The capacitors achieve automatic voltage balancing through a series/parallel connection with the input voltage source. To control the switching pulses of the switches, level-shifted pulse width modulation (LS-PWM) strategy has been employed. A comparative evaluation has been performed between the proposed structure and structures presented in recent articles, considering various parameters such as voltage gain, number of DC sources, number of semiconductor devices, maximum blocking voltage (MBV), and total standing voltage (TSV). Considering this comparison, the lower number of semiconductor devices for generating a 17-level output with suitable voltage gain, and especially the cost-effectiveness of the structure, are the main advantages of the proposed configuration. In addition, a soft charging method has been employed to limit the inrush current of capacitors. Moreover, the power losses of the proposed structure have been investigated, indicating its acceptable efficiency. Finally, for the analysis and validation of the proposed structure's performance, an experimental prototype has been implemented and evaluated under various conditions. The results indicate satisfactory performance of the proposed structure under various stable and dynamic operating conditions.

## Introduction

Multi-level inverters (MLIs) have been developed due to various advantages such as better harmonic performance, higher efficiency, and lower voltage stress on switches. Multilevel inverters are widely used for integration of renewable energy resources in low/medium-voltage grid, FACTS devices, electric vehicles, variable speed drives, smart grids, etc. The proposed topology can be used in the integration of renewable energy resources as photovoltaic systems or fuel cells to the grid or off-grid applications. The topologies presented in Refs.^1–4^ are also used for photovoltaic applications. In general, MLIs are classified into three types of topologies: flying-capacitor (FC), neutral-point clamped (NPC), and cascaded H-bridge (CHB). Each of these three conventional topologies, besides providing suitable performance, has limitations such as capacitor voltage imbalance in NPC and FC topologies and the necessity for numerous separate DC sources in CHB topology^5–7^. Therefore, the limitations of conventional MLIs for high-voltage applications lead to an increase in the cost and complication of these structures.

Switched-capacitor multi-level Inverters (SC-MLIs) are new structures that are a suitable improved alternative to conventional MLIs for overcoming the limitations of traditional structures^8^. Significant features of SC-MLIs involve generating a maximum number of output voltage levels, achieving high voltage gain without the need for bulky transformers or inductors, reducing the number of semiconductor devices, and decreasing the maximum voltage stress (MVS) on switches. However, the process of charging capacitors is one of the limitations of SC-MLIs. The significant inrush current during the charging of capacitors increases current stress on the switches involved in the charging path, which leads to a decrease in the converter's overall efficiency^9^.

Recently, several structures have been introduced for SC-MLIs. However, each structure has its own limitations along with its advantages. In reference^10^, a 13-level switched-capacitor inverter with a voltage gain of 6 is presented. This structure features automatic voltage balancing of the capacitors without the need for complex control schemes and additional auxiliary circuits, and it also does not require an H-bridge module for generating negative voltage levels. A notable feature of this structure is the use of Improved Nearest Level Modulation method to enhance the power quality of the output voltage. However, this structure requires two high-voltage capacitors. In reference^11^, a 15-level switched-capacitor inverter with a reduced number of switches is introduced. This structure achieves automatic voltage balancing of the capacitors without the need for complex control circuits. Additionally, it can be extended further to achieve higher levels. However, this structure requires two voltage sources to produce a 15-level voltage output with a voltage gain of 7. In reference^12^, a multi-level switched-capacitor inverter with two voltage sources is presented, capable of operating in both symmetrical and asymmetrical modes. This structure features automatic voltage balancing of the capacitors and does not require an H-bridge module for generating negative voltage levels. In reference^13^, a 13-level switched-capacitor structure with self-balanced capacitor voltages is presented. This structure is suitable for medium-voltage applications. Nevertheless, the required number of components for generating 13-level output with triple voltage gain is relatively high, which is undesirable. Reference^14^ presents a novel 25-level switched-capacitor structure with a double voltage gain. This structure possesses the capability of self-balanced capacitors voltage and can be extended to generate higher voltage levels. Nevertheless, the structure requires several separate DC sources. In reference^15^, a 21-level structure with the capability of self-balanced capacitors voltage has been presented. This structure is suitable for medium/high voltage applications and does not require any H-bridge module to produce negative voltage levels. Nevertheless, it requires a large number of capacitors and power switches, which leads to an increase in the size and cost. In reference^16^, a 13-level structure with sextuple voltage gain has been presented. The features of this structure include the capability of self-balanced capacitors voltage, negative voltage levels producing without any H-bridge module, and utilization of only a single DC source. However, the nine switches in this structure handle the maximum blocking voltage (MBV). In the above-studied structures, no solution has been presented to limit the inrush current. The prominent issue in the structures of the aforementioned references is the high inrush current, leading to high current stress on the switches and reduced reliability. Therefore, reducing/controlling current stress/switching losses using various methods is essential.

Some Methods have been introduced regarding the challenge of reducing inrush current during the capacitor charging SC-MLIs including soft charging methods and hybrid pulse width modulation (PWM) techniques. For instance, structures for limiting inrush current using these methods have been presented in references^17–19^. In the 17-level structure presented in reference^17^, a voltage gain of 8 is achieved using only one DC source, 12 switches, five diodes, and four capacitors. This structure, using the quasi-soft charging method for capacitors, reduces the voltage stress on devices and limits high inrush current. However, it can be noted that the total standing voltage (TSV) is relatively high, and this structure requires four high-voltage capacitors. In reference^18^, a 9-level structure with characteristics of modularity, input voltage boosting, leakage current elimination, and suitability for PV applications has been presented. This structure presents two different topologies for capacitor charging using a series inductor with an input source, diode, and switch to eliminate high inrush currents of the capacitors. In contrast, the required number of components for this structure is significantly high, leading to an increase in cost, size, and a decrease in the reliability of the structure. In the structure presented in reference^19^, utilizing an inductor in the path of capacitor charging as well as several charging diodes, the high inrush current has been limited. This structure is particularly suitable for PV applications. The charging inductor in this structure has three prominent roles: filtering input current harmonics, preventing the capacitor inrush current, and facilitating the maximum power tracking process. In contrast, this structure suffers from a large number of diodes and capacitors with high voltage ratings.

In this article, a quadruple boost 17-level switched-capacitor inverter has been proposed. The advantages of the proposed structure include utilizing only one DC source, 11 power switches, two diodes, three capacitors with self-balanced capacitors voltage, reduction in capacitor inrush current, and suitable values for TSV and MBV. The proposed structure can produce negative voltage levels without any H-bridge module.

The continuation of the article is organized as follows: The structure of the proposed inverter, including its circuit operation, capacitors design, modulation strategy, and soft charging method, is detailed in Sect. "Proposed topology". Sections "Power loss analysis" and "Comparison with other topologies" sequentially present the power losses analysis and a comparative evaluation. In Sect. "Experimental results", experimental results of the proposed structure are provided to demonstrate its feasibility and performance accuracy. Finally, the conclusion is presented in Sect. "Conclusion".

## Proposed topology

### Circuit description

**Figure 1:**
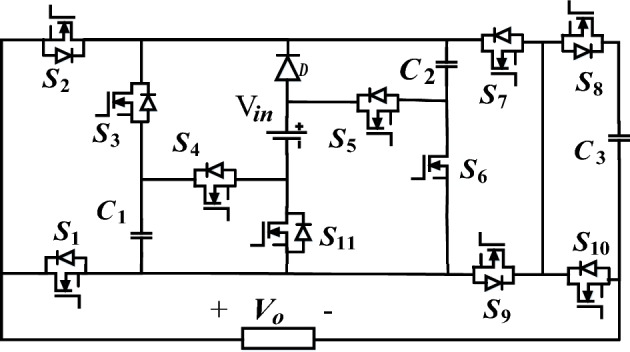
Proposed switched capacitor inverter structure.

The structure of the proposed quadruple boost 17-level switched-capacitor inverter is shown in Fig. 1. The proposed structure comprises eleven power switches (S_1_-S_11_), one diode D, three capacitors (C_1_, C_2_, and C_3_), and only one DC source (V_in_). In this structure, all three capacitors automatically achieve balance without any complex control methods or external circuits. This is attained using a series/parallel connection with the input voltage. In the proposed structure, the voltage of capacitors C_1_, C_2_, and C_3_ are automatically regulated to the values of V_in_, 2V_in_, and 0.5V_in_, respectively. In this structure, the switches S_1_ and S_2_, as well as S_8_ and S_10_, operate complementary. Due to this characteristic and the inherent voltage regulation of the capacitors, the control complexity of the proposed inverter decreases.

### Operation principle

**Figure 2:**
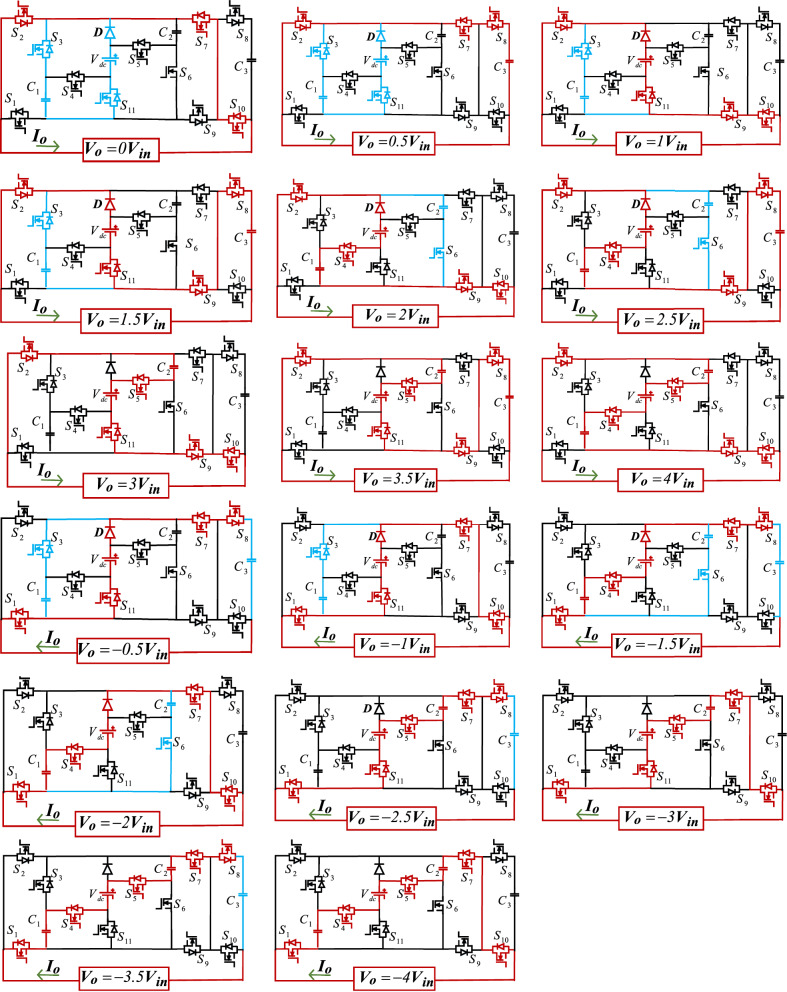
Performance of the proposed inverter in generating various voltage levels.

Different switching states of the proposed inverter are described in Table 1. The current path and operational modes for achieving various output voltage levels are depicted in Fig. 2. The charging capacitor path is shown in blue, and the discharging capacitor path and output voltage level generation are indicated in red. According to Fig. 2, to confirm the automatic balance of the capacitors and the proper operation of the proposed inverter structure, its circuit is analyzed as follows.

**Table 1:** Switching states of the proposed inverter.

Output voltage	Conducting switches	Output voltage	Conducting switches
− 0.5V_in_	S_1_, S_3_, S_7_, S_8_, S_11_	0.5V_in_	S_2_, S_3_, S_7_, S_8_
− 1V_in_	S_1_, S_3_, S_7_, S_10_, S_11_	1V_in_	S_2_, S_3_, S_9_, S_10_, S_11_
− 1.5V_in_	S_1_, S_4_, S_6_, S_7_, S_8_	1.5V_in_	S_2_, S_3_, S_8_, S_9_, S_11_
− 2V_in_	S_1_, S_4_, S_6_, S_7_, S_10_	2V_in_	S_2_, S_4_, S_6_, S_9_, S_10_
− 2.5V_in_	S_1_, S_5_, S_7_, S_8_, S_11_	2.5V_in_	S_2_, S_4_, S_6_, S_8_, S_9_
− 3V_in_	S_1_, S_5_, S_7_, S_10_, S_11_	3V_in_	S_2_, S_5_, S_9_, S_10_, S_11_
− 3.5V_in_	S_1_, S_4_, S_5_, S_7_, S_8_	3.5V_in_	S_2_, S_5_, S_8_, S_9_, S_11_
− 4V_in_	S_1_, S_4_, S_5_, S_7_, S_10_	4V_in_	S_2_, S_4_, S_5_, S_9_, S_10_
0V_in_	S_1_, S_9_, S_10_	0V_in_	S_2_, S_7_, S_10_

The capacitor C_3_ is discharged during all odd levels of the positive half-cycle and charged during all odd levels of the negative half-cycle. When capacitor C_1_ is in parallel with the DC source, it charges to V_in_, and when it is in series with the DC source, it discharges. The capacitor C_2_ is charged to a voltage of 2V_in_ when it is in parallel connection with the series of DC power source and the capacitor C_1_, and discharged in series connection with the DC power source.

### Capacitor design

**Figure 3:**
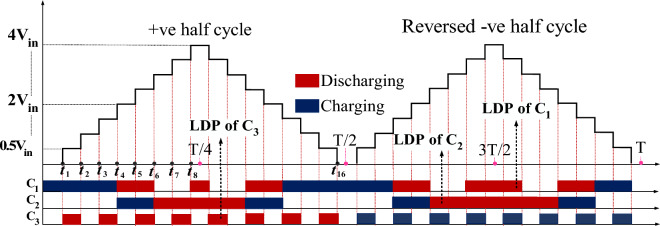
Charging and discharging process of capacitors.

In Fig. 3, the largest time intervals for charging and discharging capacitors and the 17 operational modes of the output voltage are illustrated in one cycle. Since the maximum discharge of the capacitors occurs under a purely resistive load, the capacitors design procedure is considered under a purely resistive load. Various factors such as the maximum discharge time interval of capacitors C_1_ and C_2_, the total discharge time interval of capacitor C_3_ in the positive half-cycle, capacitor voltage ripple, and the nominal frequency affect the capacitance value. Therefore, it is essential to control the capacitor voltage ripple in the proposed structure to improve voltage quality and reduce power losses. This voltage ripple is caused by the discharge of the capacitor to supply the load. The acceptable voltage ripple for the capacitors ranges from 5 to 10 percent^20^. According to Fig. 3, the discharge amount of capacitors, considering a constant time interval for different voltage levels, is expressed according to Eqs. (1) to (3).

1 $$\Delta {Q}_{C1}=2{\int }_{{t}_{7}}^\frac{T}{4}{I}_{omax}{\text{sin}}\left(\omega t-\varphi \right)dt,$$

2 $$\Delta {Q}_{C2}=2{\int }_{{t}_{5}}^\frac{T}{4}{I}_{omax}{\text{sin}}\left(\omega t-\varphi \right)dt$$

3 $$\Delta {Q}_{C3}=\left[{\int }_{{t}_{1}}^{{t}_{2}}{I}_{omax} {\text{sin}}\left(\omega t-\varphi \right)dt+{\int }_{{t}_{3}}^{{t}_{4}}{I}_{omax} {\text{sin}}\left(\omega t-\varphi \right)dt+{\int }_{{t}_{5}}^{{t}_{6}}{I}_{omax} {\text{sin}}\left(\omega t-\varphi \right)dt+{\int }_{{t}_{7}}^{{t}_{8}}{I}_{omax} {\text{sin}}\left(\omega t-\varphi \right)dt\right],$$

where *φ* is the phase difference between the load current *I*_*o*_ and the fundamental component of the output voltage, and *I*_*omax*_ is the peak value of the load current. Relation (4) illustrates the overall expression for time interval for various output voltage levels to calculate the capacitance which are indicated in (5) for each time intervals.

4 $${t}_{i}=\frac{{\mathit{sin}}^{-1}\left(\frac{2i-1}{{N}_{l}-1}\right)}{\omega },$$

5 $${t}_{1}=\frac{{\mathit{sin}}^{-1}\left(\frac{1}{16}\right)}{\omega }=0.00019 \,\,\,\,\,\,\,{t}_{2}=\frac{{\mathit{sin}}^{-1}\left(\frac{3}{16}\right)}{\omega }=0.0006,$$

$${t}_{3}=\frac{{\mathit{sin}}^{-1}\left(\frac{5}{16}\right)}{\omega }=0.0010 \,\,\,\,\,\,\,{t}_{4}=\frac{{\mathit{sin}}^{-1}\left(\frac{7}{16}\right)}{\omega }=0.00144$$

$${t}_{5}=\frac{{\mathit{sin}}^{-1}\left(\frac{9}{16}\right)}{\omega }=0.0019 \,\,\,\,\,\,\,{t}_{6}=\frac{{\mathit{sin}}^{-1}\left(\frac{11}{16}\right)}{\omega }=0.00241$$

$${t}_{7}=\frac{{\mathit{sin}}^{-1}\left(\frac{13}{16}\right)}{\omega }=0.0030189 \,\,\,\,\,\,\,{t}_{8}=\frac{{\mathit{sin}}^{-1}\left(\frac{15}{16}\right)}{\omega }=0.00387$$

$${t}_{9}=\left(\frac{T}{2}-{t}_{8}\right)=0.00614$$

where N_L_ is the number of output levels. Considering the voltage ripple relationship expressed in Eq. (6), the capacitance is formulated according to (7).

6 $$\Delta {V}_{c}=\frac{\Delta {Q}_{C}}{C}$$

7 $${C}_{1}\ge \frac{\Delta {Q}_{C1}}{K{V}_{in}} \,and \,{C}_{2}\ge \frac{\Delta {Q}_{C2}}{K\left(2{V}_{in}\right)} \,and \,{C}_{3}\ge \frac{\Delta {Q}_{C3}}{K\left(0.5 {V}_{in}\right)} ,$$

where *K* represents the allowable voltage ripple percentage of capacitors. Finally, the capacitance is determined using the above equations as follows:

8 $${C}_{1}\ge \frac{{2 I}_{omax}}{K \omega { V}_{in}}\left( \mathit{cos}\left(0.9484-\varphi \right)-\mathit{sin}\varphi \right),$$

9 $${C}_{2}\ge \frac{{ I}_{omax}}{K \omega { V}_{in}}\left( \mathit{cos}\left(0.5974-\varphi \right)-\mathit{sin}\varphi \right),$$

10 $$\begin{aligned} C_{3} & \ge \frac{{2 I_{omax} }}{{K \omega \left( {0.5 V_{in} } \right)}}[\cos \left( {0.0625 - \varphi } \right) + \cos \left( {0.318 - \varphi } \right), \\ & + \cos \left( {0.597 - \varphi } \right) + cos\left( {0.948 - \varphi } \right) \\ &+ cos\left( {0.948 - \varphi } \right) - \cos \left( {0.189 - \varphi } \right) - \cos \left( {0.453 - \varphi } \right) \\ &- \cos \left( {0.758 - \varphi } \right) - \cos \left( {1.215 - \varphi } \right)] , \\ \end{aligned}$$

### Modulation scheme

**Figure 4:**
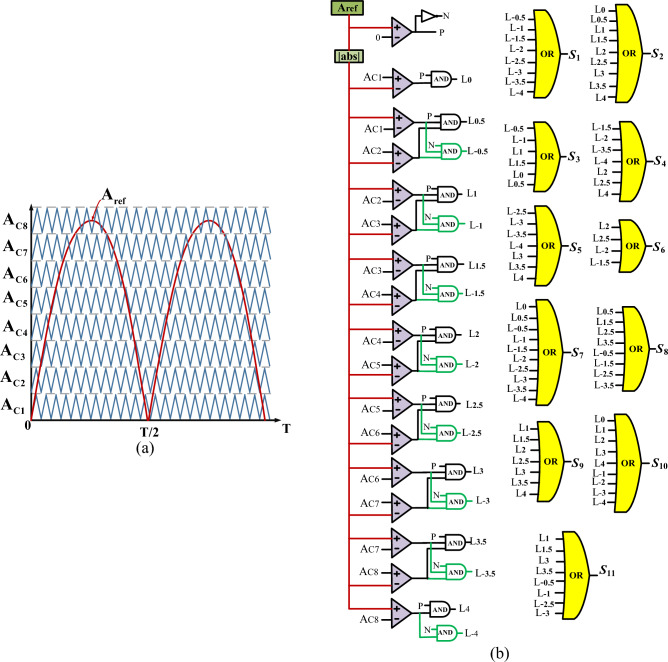
Applied LS-PWM switching scheme, (**a**) Overall view, (**b**) Implementation scheme.

Various methods introduced for the control strategy of multi-level inverters such as high-frequency switching methods like Space Vector Pulse Width Modulation (SV-PWM), Level-Shifted Pulse Width Modulation (LS-PWM), and Phase-Shifted Pulse Width Modulation (PS-PWM)^21,22^, as well as low-frequency switching methods like nearest level control and selective harmonic elimination^23,24^. Among these methods, the strategy of Level-Shifted Pulse Width Modulation (LS-PWM)^25^ is commonly employed for controlling the switching pulses of multi-level inverters. This method provides an output waveform close to sinusoidal with low harmonic content. In this article, for controlling the switches of the proposed structure, the strategy of LS-PWM has also been employed. The switching pattern of the proposed structure for generating a 17-level output is shown in Fig. 4. According to this figure, the modulation process is divided into eight sections. In each section, the reference sinusoidal waveform *A*_*ref*_ with a frequency of *f* is compared to carrier waveforms *A*_*C1*_-*A*_*C8*_ with the same amplitude and frequency of *f*_*s*_. The modulation index is expressed as $M=\frac{{A}_{ref}}{8{A}_{c}}$. Finally, the switching pulses are generated using the relationship between the sinusoidal waveform and the carrier waveforms in each section, and by applying the appropriate logic according to Table 1.

### Soft charging

**Figure 5:**
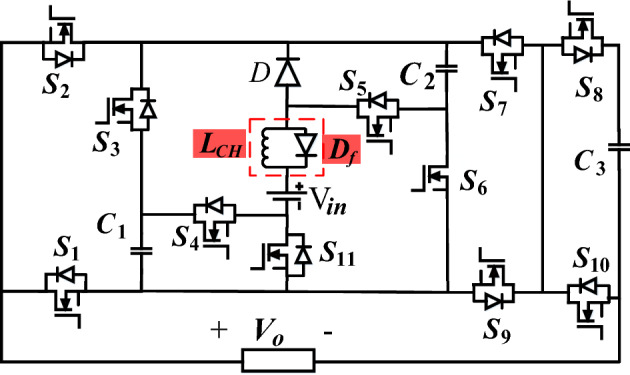
The proposed structure along with the soft charging method.

Multi-level switched-capacitor inverters experience a significant inrush current during the charging of capacitors. A high inrush current of capacitors leads to an increase in current stress on the switches associated with the charging process, capacitor and power semiconductor device failures, and a decrease in the reliability of the converter. Therefore, the capacitor charging process of SC-MLIs is of significant importance, and controlling the inrush current of the capacitors is essential. A soft charging method has been employed to reduce the inrush current in the proposed structure. In Fig. 5, the proposed structure with a soft charging unit is illustrated. In accordance with this figure, a charging inductor (*L*_*CH*_) along with a freewheeling diode (*D*_*f*_) is used in series connection with the DC input source to limit the inrush current. The inductor prevents sudden current changes and attenuates the inrush current, but it results in voltage spikes. Therefore, a diode is used in parallel with the soft charge inductor to prevent this overvoltage. This diode hinders overcharging of the capacitors and leads to steadying of the voltage of the capacitors^18^. Therefore, the presence of an inductor in the capacitor charging path enables soft charging with low current stress, enhancing the reliability of the converter.

## Power loss analysis

In this section, power loss analysis is presented to evaluate the performance of the proposed 17-level inverter. In general, three types of losses occur in multi-level inverters on power semiconductor devices and capacitors. The losses in power semiconductor switches include switching losses and conduction losses, where the losses in capacitors are capacitor ripple losses. The total power losses for the proposed 17-level switched-capacitor inverter are calculated according to Eq. (11).

11 $${P}_{losses}={P}_{c}+{P}_{sw}+{P}_{r},$$

where *P*_*sw*_, *P*_*c*_, and *P*_*r*_ represent switching losses, conduction losses, and capacitor ripple losses, respectively. Based on Eq. (11), the efficiency of the proposed inverter can be expressed as follows:

12 $$\eta =\frac{{P}_{out}}{{P}_{out}+{P}_{losses}}=\frac{{P}_{out}}{{P}_{out}+{P}_{c}+{P}_{sw}+{P}_{r}}.$$

### Switching losses

Switching losses are the total losses associated with transitions of the switch in turning on and turning off, which result from the non-ideal characteristics of power semiconductor devices. The non-ideal characteristics of power semiconductor devices during switching state transitions results in delays when the switch is turning on or off. This delay leads to switching losses. To calculate the switching losses, a linear approximation is considered for the transition in voltage and current. Therefore, the switching losses of the switches can be expressed as follows^26^.

13 $${P}_{sw}=f\left[\sum_{k=1}^{{N}_{S}}\left(\sum_{i=1}^{{N}_{ON,k}}\frac{{V}_{sw,k}\times {I}_{ON}\times {t}_{ON}}{6}+\sum_{i=1}^{{N}_{OFF,k}}\frac{{V}_{sw,k}\times {I}_{OFF}\times {t}_{OFF}}{6}\right)\right],$$

wherein *f*, *N*_*S*_, and *V*_*sw,k*_ respectively represent the output voltage frequency, the total number of switches, and the voltage across switch *k* when it is in off-state. *I*_*ON*_ and *I*_*OFF*_ represent the current flowing through the switch after the switch is turned on and before the switch is turned off, respectively. *t*_*ON*_ and *t*_*OFF*_ represent the time required to turn on and turn off a switch, respectively. *N*_*ON,k*_ and *N*_*OFF,k*_ indicate the number of times that switch *k* turns on and off in an output voltage period.

### Conduction losses

The conduction losses depend on the losses across the switch (*P*_*c,s*_) and its anti-parallel diode (*P*_*c,d*_). These losses, resulting from the parasitic parameters of semiconductor devices and their voltage drop in the on-state, are calculated as follows.

14 $${P}_{c,s}={V}_{s,ON}i\left(t\right)+{R}_{s}{i}^{\alpha }\left(t\right),$$

$${P}_{c,d}={V}_{d,ON}i\left(t\right)+{R}_{d}{i}^{2}\left(t\right),$$

where *V*_*s,ON*_ and *R*_*s*_ respectively represent the voltage drop and switch resistance when the switch is in the ON state, and similarly, *V*_*d,ON*_ and *R*_*d*_ represent the voltage drop and diode resistance during diode conduction. α is a constant coefficient dependent on the switch characteristics. The total conduction losses in all switches and anti-parallel diodes are expressed as follows^27^.

15 $${P}_{c}=\sum_{k=1}^{{N}_{s}}\frac{1}{2\pi }{\int }_{0}^{2\pi }\left[{V}_{s,ON}i(t)+{R}_{s}{i}^{\alpha }(t)\right]dt+\sum_{k=1}^{Nd}\frac{1}{2\pi }{\int }_{0}^{2\pi }\left[{V}_{d,ON}i(t)+{R}_{d}{i}^{2}(t)\right]dt.$$

### Ripple losses

The ripple losses occur due to the voltage difference between the source voltage and the desired capacitor voltage. When capacitors are charged in parallel with a DC source, the equivalent series resistance (ESR) of the capacitor in the charging loop results in this voltage difference. In fact, a voltage ripple appears across the capacitor which directly affects the ripple losses. Therefore, ripple losses in the capacitor are expressed by the following equation^28^.

16 $${\Delta V}_{{c}_{i}}=\frac{1}{{C}_{i}}{\int }_{{t}_{a}}^{{t}_{b}}{I}_{c,i}\left(t\right) dt,$$

$${P}_{r}=\frac{1}{2}{f}_{sw} \sum_{i=1}^{N}{C}_{i} \Delta {{V}_{{C}_{i}}}^{2},$$

where *N*, *I*_*C,i*_, and *t*_*b*_*-t*_*a*_ represent the number of capacitors, capacitor charging current, and the duration of capacitor discharge, respectively.

## Comparison with other topologies

In this section, several structures of SC-MLLs have been compared using different and varied indicators to analyze the advantages and disadvantages of the proposed inverter. In Table 2, the 17-level proposed structure has been compared to references^17,20^ and Refs.^29–34^ based on various parameters such as voltage gain, the number of DC sources, the number of semiconductor devices, maximum Blocking Voltage (MBV), and Total Standing Voltage (TSV). Furthermore, the cost function CF^17^ has also been employed to evaluate compared topologies. This cost function can be calculated based on the following relationship:

17 $$CF=\left({N}_{SW}+{N}_{Dr}+{N}_{DD}+{N}_{C}+({\mathrm{\alpha }}_{1}TSV/B\right))\times {N}_{DC}/{N}_{Level},$$

where α_1_ is a weighting factor indicating the importance of the number of converter components or the TSV value. If the designer's intention is to have fewer components, this factor is considered 0.5. While if the designer's intention is a structure with fewer TSV, this coefficient is considered 1.5^35^. According to Table 2, the proposed inverter with a weighting factor of 0.5 and also a weighting factor of 1.5 has a lower cost compared to most other comparative structures.

**Table 2:** Comparison of 17-level SC-MLIs.

Topologies	N_SW_	N_DD_	N_Dr_	N_C_	N_DC_	B	TSV/B	MBV	CF α = 0.5	CF α = 0.5	SUF
^17^	12	5	12	4	1	8	6.13	4	2.12	2.48	0.04
^20^	14	2	14	3	1	8	5.9	4	2.11	2.46	0.08
^29^(a)	10	4	9	6	1	2	5.75	2	1.88	2.21	0.098
^29^(b)	10	4	9	6	1	4	5.75	4	1.88	2.21	0.069
^30^	13	6	13	4	1	2	6.25	2	2.30	2.68	0.03
^31^	12	0	9	4	2	2	6.25	2	3.54	4.27	0.11
^32^	11	2	11	2	2	2	6.75	2	3.25	3.87	0.13
^33^	12	2	12	3	1	4	6.75	4	1.90	2.30	0.04
^34^	14	4	14	4	1	8	6.38	4	2.31	2.68	0.13
Proposed topology	11	1	11	3	1	4	7	4	1.73	2.14	0.17

In multilevel converters, a higher number of switches are used to achieve higher voltage levels and power, as well as to generate more voltage levels and improve the quality of the output voltage. Consequently, the semiconductor utilization factor (SUF) in these converters is lower compared to dc-dc converters. The semiconductor utilization factor, which includes the utilization of the switches and diodes, is expressed using the following equation^36^:

18 $$SUF={P}_{o}/ \sum_{j=1}^{K}{V}_{Sj}{I}_{Sj},$$

where ${V}_{Sj}$ and ${I}_{Sj}$ represent the maximum voltage and RMS current of semiconductor devices, respectively, and *K* is the number of semiconductor devices. The SUF should be maximized to reduce the cost of semiconductor devices utilized in the circuit. As mentioned in Ref.^36^, in a well-designed converter, the voltage and current across a semiconductor device are minimized while maximizing the output power. By comparing the SUF values for the proposed structure with those of comparative structures, it is evident that the proposed structure offers a higher SUF value than others. This implies a lower cost of power electronic devices per specified output power for the proposed structure. The analysis of the SUF parameter yields similar results to parameters CF_1_ and CF_2_, indicating that based on all three cost-related parameters, the proposed structure offers lower costs compared to recently proposed structures.

In the structure presented in reference^17^, a higher voltage gain has been achieved using more switching devices compared to the proposed structure. This structure requires an H-bridge module to generate AC output voltage. An increased number of switching devices and H-bridge module results in higher costs and converter losses. Reference^30^ provides a better TSV and MBV compared to the proposed structure and only two switches tolerate the maximum blocking voltage. However, it requires a large number of components to achieve double voltage gain, which makes it less desirable. In references^31,32^, although they offer fewer TSV compared to the proposed structure, they require more sources to generate a voltage gain of two, which is not cost-effective. The proposed topology, with structural similarity and TSV closeness to the topology presented in reference^33^, offers fewer switches. The topology presented in reference^34^ provides twice the voltage gain compared to the proposed structure. However, the number of its components is significantly higher, and it includes eight switches that tolerate the maximum output voltage which results in its higher cost.

The total number of components in the proposed structure is fewer in comparison to the studied 17-level structures. Although the proposed structure has the identical count of switches as the structure in Ref.^32^, it offers only the voltage gain of 2 with two DC sources. Therefore, the proposed structure offers advantages such as a simple design, a single DC source, appropriate TSV and voltage gain with a reduced number of components, making it cost-effective compared to recently studied structures.

**Figure 6:**
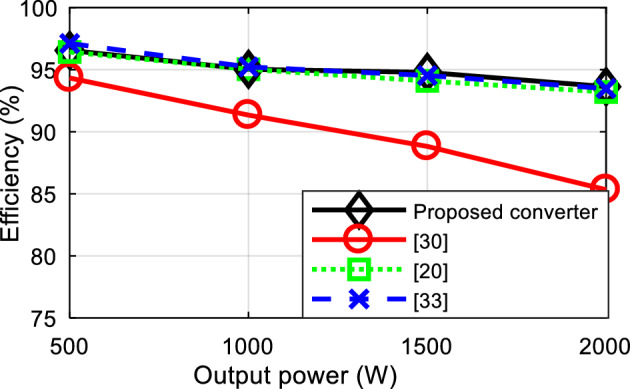
Efficiency comparison of the proposed structure with the reference structures.

Finally, the power losses of the proposed structure and reference structures^20,30,33^ are simulated for various powers, as illustrated in Fig. 6. For the analysis and comparison of losses, the structures under comparison have been simulated under completely identical conditions to enable a fair comparison in terms of losses and efficiency. Considering Fig. 6, it can be stated that the proposed structure, in comparison to the structures under evaluation, provides equal or better efficiency, especially at higher powers.

## Experimental results

**Figure 7:**
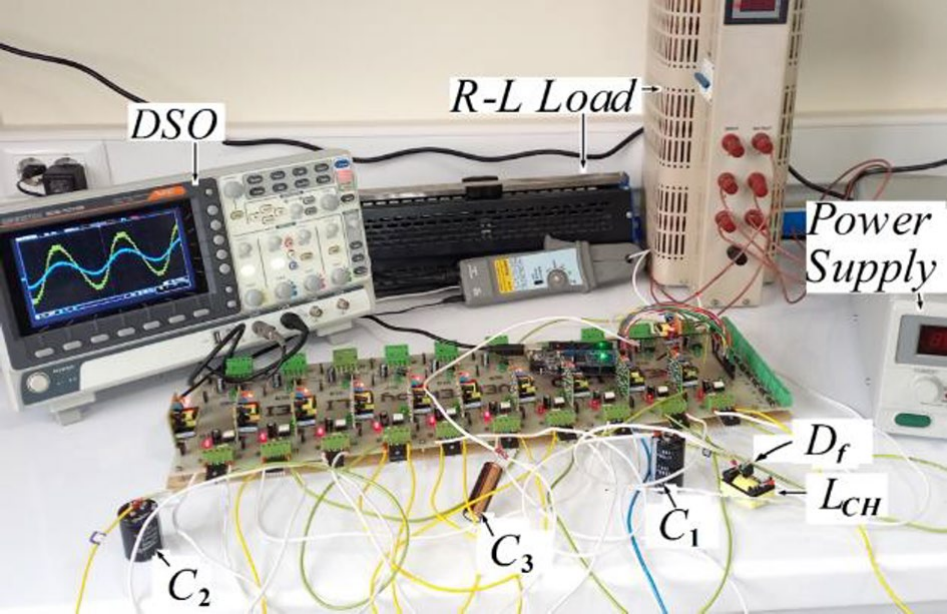
Experimental prototype of the proposed inverter.

In order to analyze and validate the performance of the proposed structure, a laboratory prototype has been implemented on a 500W scale, as illustrated in Fig. 7. This circuit includes a 70 V DC source as the input voltage, a 0.1 mH inductor along with a freewheeling diode to limit the inrush current, the diode, and switches with specifications of MBR20B200 and IRFP460, respectively. A microcontroller is employed to generate switching pulses using the pulse width modulation (PWM) technique. In Table 3, the required specifications for implementation are presented. The capacitors value used in the circuit has been calculated based on the relationships provided in Eqs. (8) to (10).

**Table 3:** The specifications for implementation.

Parameter	Value
70V	Input voltage (V_in_)
0.94	Modulation index
50HZ	Fundamental frequency
3500 HZ	Switching frequency
R = 50 Ω, R-L = 50Ω–100 mH	Load
0.1 mH	Charging inductor (L_CH_)
3300µF (100V)	Capacitor C_1_
2200µF (150V)	Capacitor C_2_
3300µF (63V)	Capacitor C_3_

**Figure 8:**
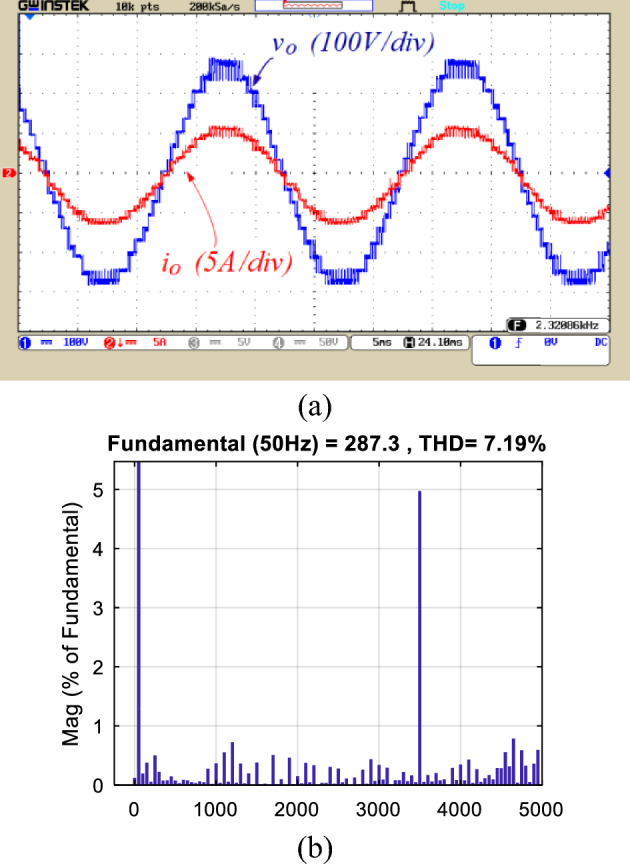
(**a**) Output voltage and load current waveforms under pure resistive loading, (**b**) THD of the output voltage.

**Figure 9:**
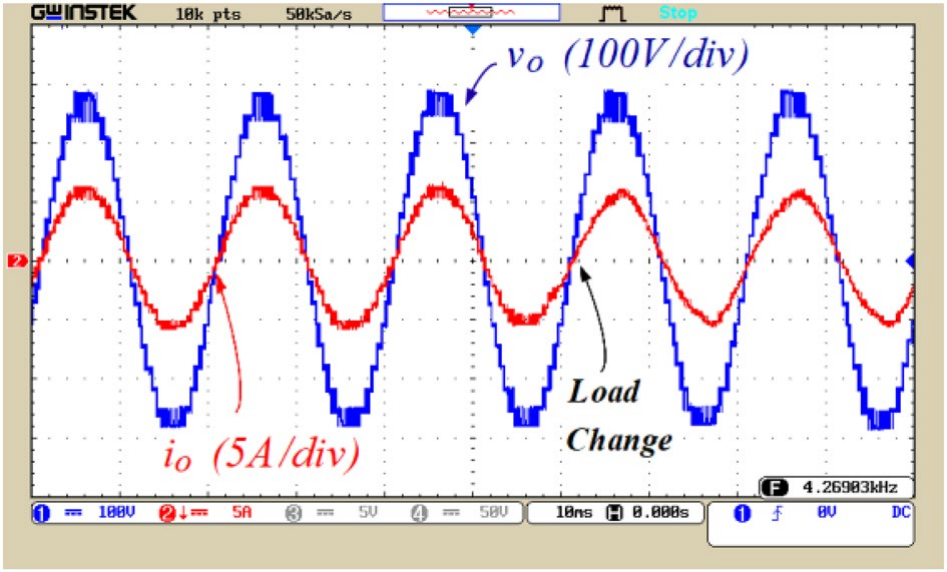
Waveforms of output voltage and load current under varying load conditions from resistive to inductive.

**Figure 10:**
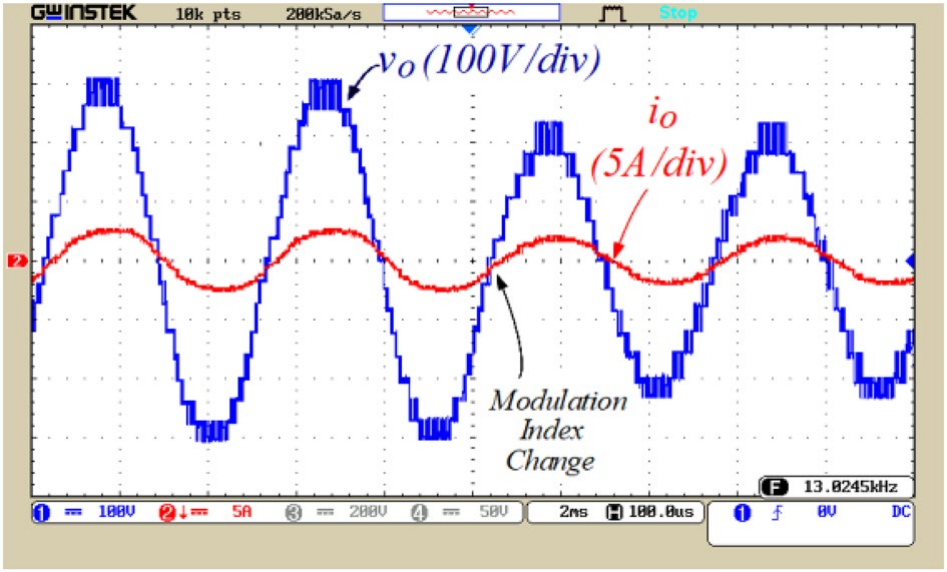
The output voltage and load current under dynamic change conditions.

**Figure 11:**
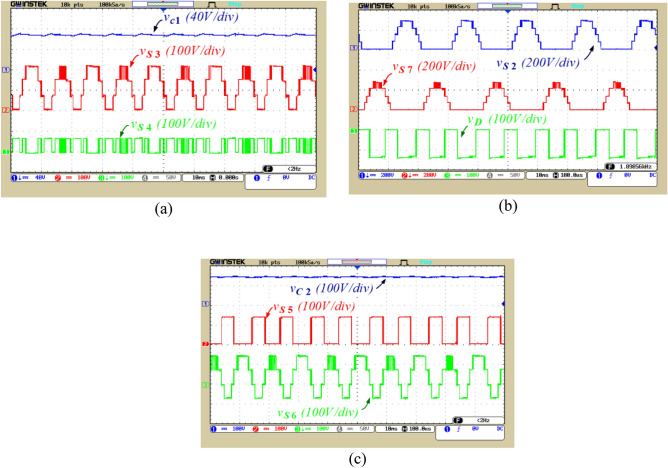
The voltage of capacitors and switches, (**a**) Voltage of C_1_, S_3_ and S_4_, (**b**) Voltage of S_2_, S_7_ and D, (**c**) Voltage of C_2_, S_5_ and S_6_.

**Figure 12:**
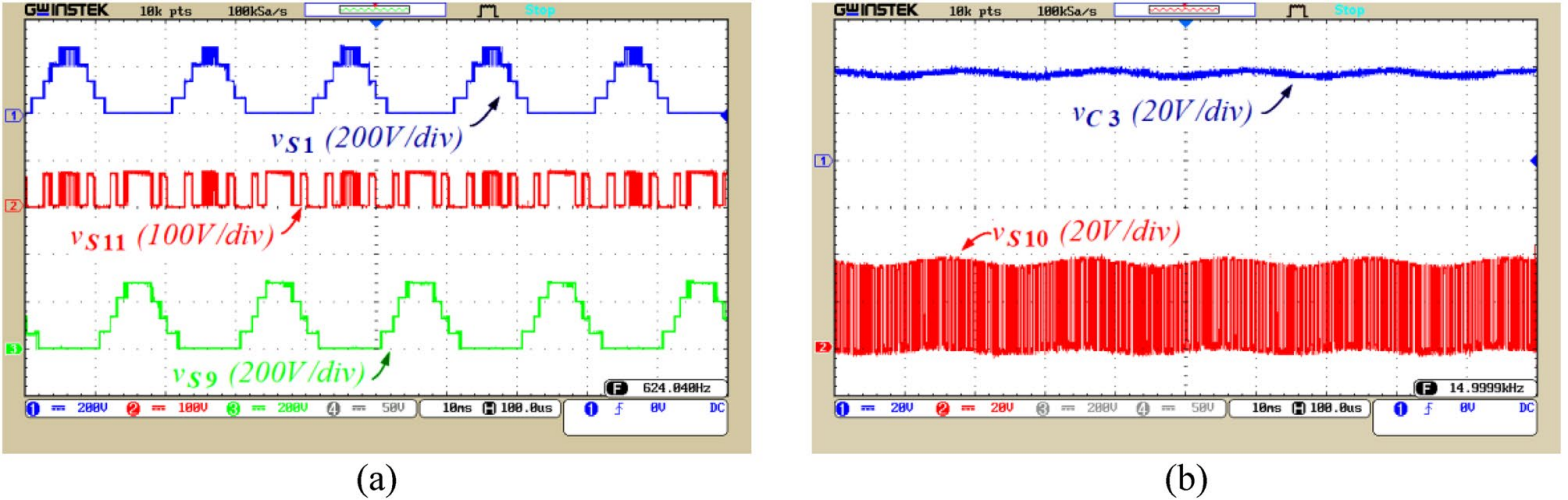
The voltage of capacitors and switches, (**a**) Voltage of S_1_, S_11_, and S_9_; (**b**) Voltage of C_3_ and S_10_.

**Figure 13:**
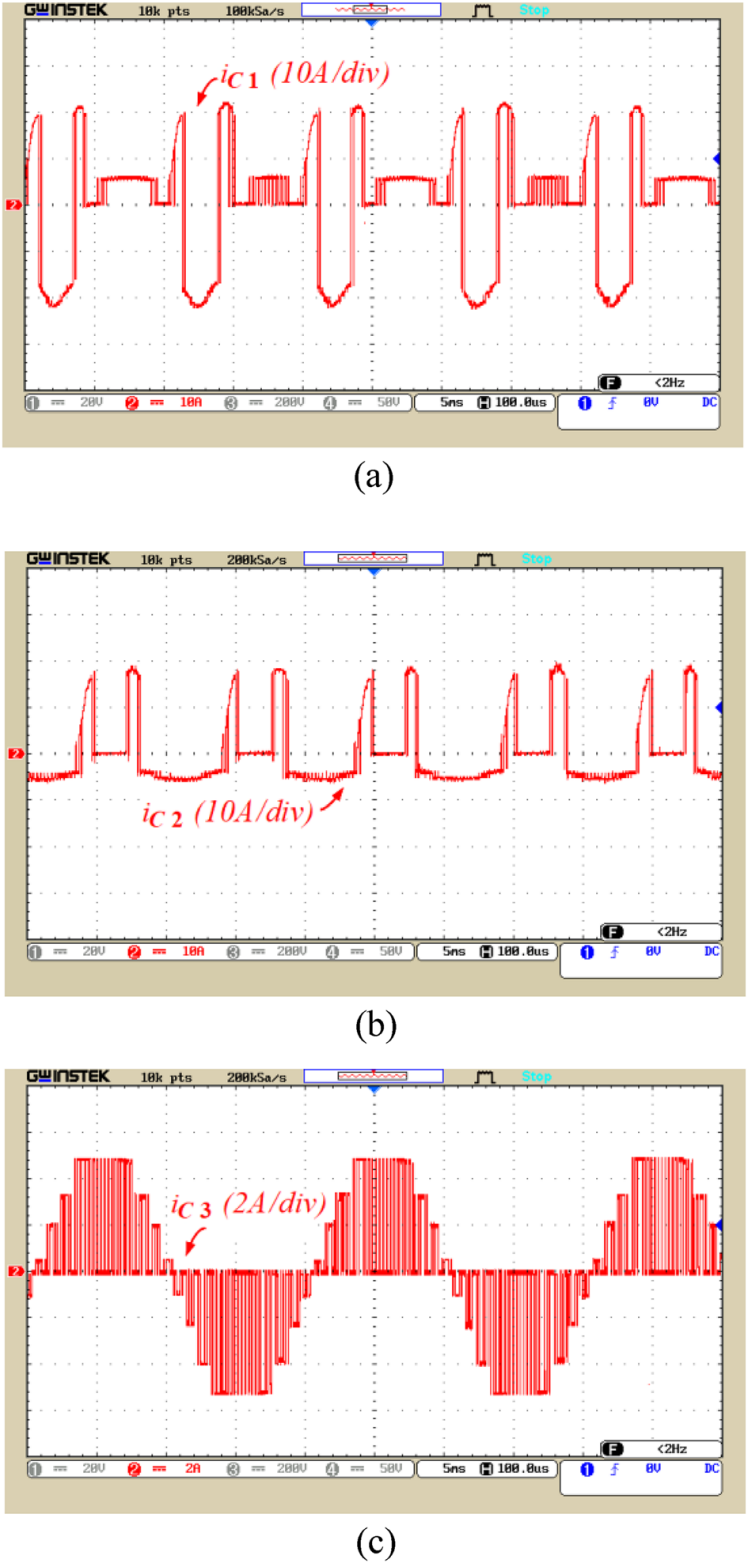
The current waveform of capacitors, (**a**) The current of C_1_, (**b**) The current of C_2_, (**c**) The current of C_3_.

**Figure 14:**
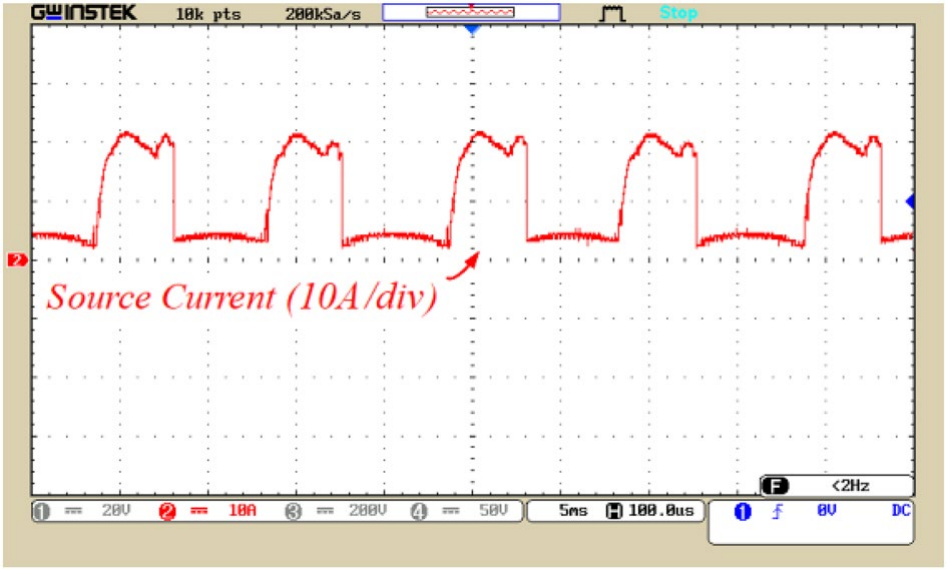
The current waveform of the input voltage source.

Figures 8, 9, 10, 11, 12, 13 and 14 display the experimental results of the proposed structure under different conditions. Figure 8 illustrates the output voltage and load current waveforms under a pure resistive load. According to Fig. 8a, the proposed inverter produces a peak output voltage of 280 V using an input voltage of 70 V. Therefore, the quadruple voltage gain 17-level output voltage is confirmed according to Fig. 8. Figure 9 depicts the results of load variation from a pure resistive load to an inductive load. The waveforms of the output voltage and load current, as shown in Fig. 9, are accurately obtained at the moment of load change and afterward. Therefore, based on Figs. 8 and 9, it can be concluded that the proposed inverter performs correctly under various power factors. Also, the value of the Total Harmonic Distortion (THD) of the output voltage is shown in Fig. 8b.

To illustrate the dynamic performance of the proposed inverter, the modulation index is varied from 0.94 to 0.70, resulting in a reduction of the output voltage levels from 17 to 13 levels, according to Fig. 10. So, by modifying the modulation index as shown in Fig. 10, the proposed inverter exhibits correct performance. Figure 11a illustrates the voltage of capacitor C_1_ and the voltage across switches S_3_ and S_4_. According to this figure, the voltage ripple of capacitor C_1_ is 4.5 V, which is equivalent to 6% of this capacitor’s voltage. Figure 11b displays the voltage across switches S_2_ and S_7_, and voltage across diode D. Figure 11c shows the voltage of capacitor C_2_ and the voltage across switches S_5_ and S_6_. According to this figure, the voltage ripple of capacitor C_2_ is 9 V, equivalent to 6.5% of this capacitor’s voltage.

Figure 12a illustrates the voltage across switches S_1_, S_11_, and S_9_. Figure 12b displays the voltage of capacitor C_3_ and the voltage across switch S_10_. According to this figure, the voltage ripple of capacitor C_3_ 2.5 V, which is equivalent to 6.5% of this capacitor’ voltage. It can be observed from Figs. 11 and 12 that the voltage of capacitors is balanced in their designed values and the ripple voltage of capacitors is not higher than the allowable limit. Moreover, considering Figs. 11 and 12, it can be stated that 4 switches (S_1_, S_2_, S_7_, and S_9_) operate at the fundamental frequency, leading to a reduction in the power losses.

The current of the capacitors and the source current with the application of the soft charging are illustrated in Figs. 13 and 14, respectively. Figure 13a andb currents flowing through capacitors C_1_ and C_2_, respectively. According to these figures, the peak current through C_1_ and C_2_ is 21 and 20 A, respectively, which confirms the limitation of the inrush current by soft charge method. Figure 13c displays the current in capacitor C_3_. According to this figure, the peak current in capacitor C_3_ is 5 A, which is significantly lower compared to the current in the other two capacitors. This is due to placement of capacitor C_3_ series with the load, which results in restricting the peak current of capacitor C_3_. Figure 14a illustrates the current flowing the input voltage source. According to Fig. 14, the peak source current is 22 A, indicating the correct performance of the soft charging method.

**Figure 15:**
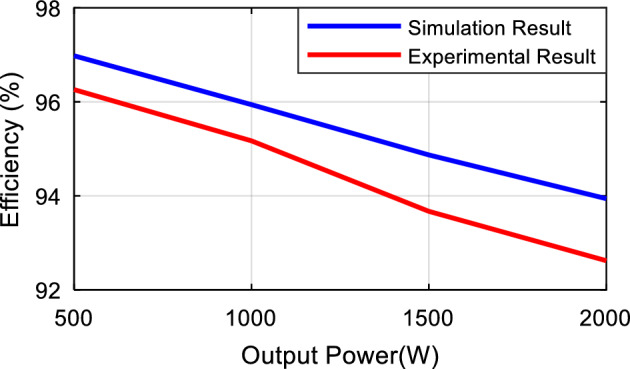
Comparison between simulation and experiment efficiencies.

By measuring the input and output power of the inverter through its voltage and current, we can calculate the losses and efficiency of the proposed structure experimentally. Using simulation, the efficiency of the proposed structure has been calculated to be 96.93% for a power of 500 watts, while its power losses reaches to 15.58 watts. Under laboratory conditions and with the same parameters, for a power of 520 watts, the losses of the proposed structure are 17.37 watts, resulting in an efficiency of 96.26%. By using simulation, the efficiency of the proposed structure for a power of 1000 watts is calculated to be 95.94%, while its power losses reaches to 42.31 watts. Under laboratory conditions for a power of 980 watts, the losses of the proposed structure are 54.23 watts, and its efficiency is 95.17%. Using simulation, the efficiency of the proposed structure for a power of 1500 watts is 94.87%, with power losses of 81.11 watts. Under laboratory conditions for a power of 1620 watts, the losses of the proposed structure are 109.04 watts, and its efficiency is 93.67%. Finally, for a power of 2000 watts, using simulation, the efficiency of the proposed structure is 93.94%, with power losses of 129.01 watts. Under laboratory conditions and with the same conditions for a power of 2020 watts, the losses of the proposed structure are 160.09 watts, and its efficiency is 92.62%. A comparison of the simulation power loss analysis with experimental efficiency results demonstrates a good correlation according to Fig. 15.

## Conclusion

In this paper, a switched-capacitor multi-level inverter is proposed, which generating a 17-level output with a quadruple voltage gain. The proposed structure includes a DC source, 11 power switches, 1 diode, and 3 capacitors. All three capacitors have the capability of self-balancing voltage without the need for complex control methods or peripheral circuits. Comparative evaluation of proposed topology with several 17-level structures presented in recent papers considering different parameters has been discussed. The proposed structure offers fewer semiconductor devices with appropriate voltage gain, and introduces a simpler and more cost-effective design considering the cost function. The cost function of the proposed structure has improved by at least 9% with a weighting factor of 0.5 and a minimum of 7% with a weighting factor of 1.5 compared to the recently presented structures. Meanwhile, the analysis of losses in the proposed structure has been investigated, and its satisfactory efficiency is confirmed compared to similar structures. To validate the performance of the proposed inverter, a laboratory prototype has been implemented. The experimental results under various conditions confirm the proper performance of the proposed structure. According to the laboratory results, the self-balanced voltage of capacitors has been maintained during load variations and even changes in modulation index.

## Data Availability

All data generated and analysed during the current study are available from the corresponding author on reasonable request.
